# Chronic pain is a risk factor for incident Alzheimer’s disease: a nationwide propensity-matched cohort using administrative data

**DOI:** 10.3389/fnagi.2023.1193108

**Published:** 2023-09-28

**Authors:** Nadège Bornier, Aurélien Mulliez, Chouki Chenaf, Antoine Elyn, Sarah Teixeira, Nicolas Authier, Célian Bertin, Nicolas Kerckhove

**Affiliations:** ^1^Service de Pharmacologie Médicale, Direction de la Recherche Clinique et de L’Innovation, Université Clermont Auvergne, Clermont-Ferrand, France; ^2^Institut Analgesia, Clermont-Ferrand, France; ^3^Centre D’Évaluation et de Traitement de la Douleur, Service de Neurochirurgie, Pôle Neuroscience, Centre Hospitalier Universitaire de Toulouse, Toulouse, France

**Keywords:** pain, Alzheimer disease, epidemiology–analytic (risk factors), incidence, dementia

## Abstract

**Introduction:**

Chronic pain (CP) is one of the most disabling conditions in the elderly and seems to be a risk factor for the development of Alzheimer’s disease and related dementias (ADRD). Only one study, using national administrative health databases, assessed and demonstrated that chronic pain (all types of pain) was a risk factor for dementia, but without assessing the impact of pain medications.

**Method:**

To assess the impact of all types of chronic pain and the long-term use of pain medications on the person-years incidence of ADRD, a retrospective nationwide healthcare administrative data study was performed using the national inter-regime health insurance information system (SNIIRAM) to the French national health data system (SNDS). Incident people >50 years old with chronic pain, defined by at least 6-months duration analgesics treatment or by a diagnosis/long-term illness of chronic pain between 2006 and 2010, were included. Chronic pain individuals were matched with non-CP individuals by a propensity score. Individuals were followed up from 9 to 13 years to identify occurrences of ADRD from 2006.

**Results:**

Among 64,496 French individuals, the incidence of ADRD was higher in the chronic pain population than control (1.13% vs. 0.95%, *p* <0.001). Chronic pain increases the risk of ADRD (HR = 1.23) and the incidence of ADRD was higher for women and increased significantly with age.

**Discussion:**

Our study highlights the importance of prevention, diagnosis, and management of chronic pain in elderly to reduce the risk of development and/or worsening of dementia.

## Introduction

Alzheimer’s disease and related dementias (ADRD, including vascular dementia, Lewy body dementia, and frontotemporal disorders according to NIH definition) are responsible for approximately 80% of diagnosed dementia cases. In 2015, the number of people affected by dementia worldwide was estimated to be nearly 47 million, and this number is expected to rise to 75 million by 2030 and 131 million by 2050, due to the aging trend in the global population (World Health Organization, 2015). In 2019, the global economic burden of ADRD was estimated at $2.8 trillion. This burden is expected to increase to $4.7 trillion in 2030 and $16.9 trillion in 2050 (Nandi et al., 2022). The care of people with dementia also impacts several sectors of society, the social care and informal care sectors even more so than direct medical care (Wimo et al., 2013). Thus, ADRD should be considered a global public health priority (World Health Organization, 2015).

There is no cure for ADRD and current medical management only slows its progression. Non-pharmacological approaches are favored in an attempt to preserve the patient’s autonomy and cognitive functions, and to adapt the environment to the loss of autonomy and cognitive disorders (Zucchella et al., 2018). Physical activity has also been shown to delay the onset of dementia (Grasset et al., 2017). From the standpoint of pharmacological treatment, four specific ADRD drugs are mainly used (donepezil, galantamine, rivastigmine, memantine; Birks, 2006), but their medical service rendered deemed insufficient. This lack of effective treatment means that medical management is mainly based on prevention, and the identification and management of risk factors (Silva et al., 2019; Zhang et al., 2021).

Chronic pain was one of the top five causes of disability (GBD 2016 Disease and Injury Incidence and Prevalence Collaborators, 2017), especially in the elderly. This prevalence can be as high as 50% among community-dwelling seniors and as high as 80% among institutionalized people (Cravello et al., 2019). Chronic pain and ADRD share common risk factors, such as advanced age, genetic impairment, depressive disorders, diabetes, obesity, social isolation, and low level of education (Van Hecke et al., 2013; Armstrong, 2019). Moreover, alterations of the nervous system are common to both pathologies, such as abnormalities of the noradrenergic system (Hayashida and Obata, 2019), overactivation of microglia and central neuroinflammation (Salter and Stevens, 2017). Certain brain areas of the “pain matrix” (the amygdala, thalamus, prefrontal cortex and hippocampus), are also involved in the development of ADRD (Willis and Westlund, 1997). These data may explain why pain has been shown to be related to dementia or cognitive decline (Whitlock et al., 2017; Khalid et al., 2020; Kao et al., 2021; Rouch et al., 2021; Wang and Liu, 2021). Nevertheless, these studies did not assess all types of pain and/or related treatments and some lack power and representativeness.

To provide additional data, we propose a larger study using national administrative health databases and by specifically assessing the impact of all chronic pain on the incidence of ADRD, and explore the impact of the long-term use of pain medications.

## Materials and methods

### Regulatory and ethics

This study was approved by the “*Centre d’épidémiologie sur les causes médicales de décès (CépiDc)–Institut national de la santé et de la recherche médicale* (INSERM),” the French data protection authority (“*Commission Nationale de l’Informatique et des Libertés*”-CNIL), and by the local Ethics Committee (IRB00013412, “CHU de Clermont-Ferrand IRB #1,” IRB number 2022-CF039) with compliance to the French policy of personal data protection.

### Data source

The data were obtained from the EGB (“*Echantillon généraliste des Bénéficiaires*”) databases created in 2005 and incorporating several databases such as reimbursements and consumption of care, hospitalizations and consultations in hospitals and specialized medical units, and long-term illness (LTI). The EGB databases represent 1/97th of the population covered by the French national health insurance system (~99% of the French population), i.e., about 700,000 individuals (Bezin et al., 2017).

### Matching and control population

A control population (without chronic pain) and without dementia history or disorders, was generated by a 3:1 (3 controls for 1 chronic pain) propensity score matching a caliper of 0.005 (using the “PROC PSMATCH” procedure on the SAS Enterprise Guide software). The propensity score incorporated age, sex, comorbidity index [Charlson score, incorporating several chronic disease (Charlson et al., 1987) see Supplementary material for details], and inclusion date (called “index date” as start of follow-up identical to matching individual with chronic pain). The absence of chronic pain was determined by the absence of a diagnosis of chronic pain and the absence of continuity of analgesic treatment for more than 3 months (all analgesics combined).

### Study population: chronic pain patient identification

A chronic pain condition was identified over the period 2006–2010 by the Anatomical Therapeutic Classification (ATC) code of recommended treatment for chronic pain (N02, M01, M02A, N03AX12, N03AX16, N06AA04, N06AA09, N06AX16, N06AX21, N01BB02, and N01BB52), with at least 6 months duration of continuous analgesic prescription (all analgesics combined), or by the medical diagnoses of chronic pain according to the International Classification of Disease (ICD-10-R521, R522, M797) or by the LTI for chronic pain (ICD-10 - R521, R522, R529, M00-M09, M10-M19, M255, M353, M40-M49, M50-M54, M79, M890, G43, and G442), as published previously (Chenaf et al., 2018; Delorme et al., 2021). To avoid chronic pain misidentification, patients with a history of mental health disorders (ICD-10 codes F00 to F99) or epilepsy (G40 and G41) were excluded from the analyzes since antidepressants or antiepileptic drugs, used as first and second line analgesics in chronic pain, are also commonly used in these neurological disorders. For code details, see Supplementary material. All individuals aged 50 years and older with chronic pain between 01/01/2006 and 12/31/2010 were included.

Chronic pain had to be incident (no diagnosis or 6-month continuous analgesic treatment before inclusion). The index date was defined as the date of chronic pain onset (diagnosis or end of the first 6-month continual period of analgesic treatment) starting the follow-up of individual. To avoid an immortality bias due to the 6-month period of analgesic treatment, control individuals who developed ADRD during the first 6 months of follow-up were excluded and replaced (matching was rerun until a ratio of 3 controls to 1 case was reached for all individuals analyzed).

### Study population: ADRD patient identification

An ADRD condition was identified over the period 2006–2019 by the ATC code of recommended treatment for dementia (N06DA and N06DX) or medical diagnoses according to the ICD-10 or LTI for ADRD (F00, F01, F03, G30, G310, G311 and G319), as published previously (Rochoy et al., 2019b; Elyn et al., 2022). For code details, see Supplementary material. To confirm the presence of ADRD, individuals had to have at least two different dates of diagnosis, or two dispensations of anti-dementia treatment, or have a diagnosis associated with a dispensation of anti-dementia treatment. Diagnosis codes of “dementia with known cause” (F02), such as Parkinson’s disease and toxic dementias, were not considered related to ADRD and are analyzed as comorbidities. Finally, the duration of follow-up was distinguished in three cases:

(i). “Onset of ADRD” = the time between inclusion and onset of ADRD,
(ii). “Death” = the time between inclusion and death,
(iii). “No event” = the time between inclusion and last follow-up.

### Assessment of exposure to pain medications

Pain medication exposure was assessed with dispensation claims recorded in the EGB database. All recommended treatments for chronic pain were considered during the study period. Exposure was described according to cumulative dose, combining both the duration of treatment and the somewhat variable daily dose, for each person, each molecule and each therapeutic class. The cumulative dose was computed during the study time window and converted it into a number of defined daily doses (DDDs) by dividing it by the average recommended daily dose for this product, as previously published by Billioti de Gage et al. (2014). Therefore, one DDD corresponded to an average one-day exposure. Exposure was ascertained up to 13 years follow-up (maximum duration of follow-up) before the onset of ADRD. Cumulative doses were categorized according to level of exposure (null = 0 DDD; mild: DDDs <180 days; moderate: DDDs ≥180 days and ≤ median distribution; and strong: DDDs > median distribution). The median distribution of each molecule and therapeutic class was calculated among people with DDDs ≥180 days. People without any claim for pain medications during the study time window served as references for the analyzes. Therapeutic classes corresponded to “NSAIDs”(M01A), “analgesics” (N02B), “antirheumatic”(M01B and M01C), “antiepileptics” (N03AX12 and N03AX16), “Serotonin-Norepinephrine Reuptake Inhibitors - SNRI”(N06AX16 and N06AX21), “TriCyclicAntidepressant - TCA”(N06AA04 and N06AA09), “triptan” (N02C) and “opioids”(N02A). Treatments used topically and only in medical settings were not included in this analysis.

### Statistics

After case identification and characterization (gender, year of chronic pain onset, age at onset, and Charlson score at onset), controls were selected using 3 nearest neighbor propensity score matching. The propensity score was computed using logistic regression with the group (case / control) as dependent variable, and gender, index date, age and Charlson score as independent variables. Groups were described after matching using frequency and associated percentage for categorical data and mean ± standard deviation, median, interquartile range and minimum-maximum for continuous data (Table 1). The person time incidence rate of ADRD (number of patients developing ADRD / follow-up time) is presented by groups (cases vs. controls) according to gender and age category in Table 2. To take into account the heterogeneity of follow-up durations, we analyzed ADRD onset using survival methods. The event of interest was defined as the onset of ADRD. Because of the advanced age of our study population and thus the significant risk of death, the latter was considered as a competitive event. Time to event was computed as the time from index date to event or latest follow-up (censor). Survival curves were plotted using the Kaplan–Meier method for crude survival (death), cumulative incidence (ADRD + death), and development of ADRD with death as a competing risk (as defined by Fine and Gray; Fine and Gray, 1999). In the univariate analysis, Log-rank and Gray’s tests were used to compare survival curves between chronic pain and control populations. In the multivariable analysis, an adjusted Fine and Gray regression model was performed to assess the risk of ADRD development (with death as competing event), adjusted on factors that were selected upon their clinical relevance and their statistical significance in univariate analysis. Moreover, collinearity between covariate candidates was carried out and in the case of high collinearity, a clinically driven decision was made to choose the covariate to be kept in univariate and multivariable models. This was the case in our study for opioid and NSAID consumption. Thus, only age, sex, hypertension, diabetes, hyperlipidemia, depression, stroke, head injury, Parkinson’s disease, epilepsy, dementia with known cause, and benzodiazepines use were selected for adjustment in the multivariable model. The main factor of interest (chronic pain) was also adjusted, considering groups (chronic pain vs. controls) that were matched using propensity score (see above).

**Table 1:** Characteristics of chronic pain and control populations.

	Chronic pain *N* = 13,596	Control *N* = 40,788
Follow-up (years)		
Mean ± SD (min-max)	9.2 ± 3.4 (0.08–13.9)	9.5 ± 3.5 (0.08–13.9)
Median (Q1-Q3)	10.2 (7.8–12.2)	10.2 (9.1–12.2)
Age (years); Mean ± SD (min-max)	65.6 ± 10.7 (50–102)	65.6 ± 10.7 (50–102)
Age groups (*n*, %)		
50–59 years	4,964 (36.5)	14,892 (36.5)
60–69 years	3,731 (27.4)	11,193 (27.4)
70–79 years	3,221 (23.7)	9,663 (23.7)
80–89 years	1,494 (11.0)	4,482 (11.0)
≥ 90 years	186 (1.4)	558 (1.4)
Female sex (*n*, %)	8,386 (61.7)	25,158 (61.7)
Comorbidities (*n*, %)		
Vascular and cerebrovascular diseases	623 (4.6)	1,793 (4.4)
Cardiac disorders	502 (3.7)	1,378 (3.4)
Cancers	1,053 (7.7)	3,832 (9.4)
Diabetes	570 (4.2)	930 (2.3)
Kidney diseases	92 (0.7)	225 (0.6)
Chronic lung diseases	465 (3.4)	994 (2.4)
Paraplegia and hemiplegia	99 (0.7)	239 (0.6)
Liver diseases	138 (1.0)	534 (1.3)
Connective tissue or rheumatic diseases	36 (0.3)	80 (0.2)
Peptic ulcers	65 (0.5)	196 (0.5)
HIV	7 (0.1)	48 (0.1)

**Table 2:** Alzheimer’s disease and related dementia incidence rates by age and sex.

	Male				Female			
	Chronic pain		Control		Chronic pain		Control	
	*n*	Incidence*	*n*	Incidence	*n*	Incidence	*n*	Incidence
All	5,210	9.2	15,630	7.2	8,386	12.5	25,158	10.9
50–54	913	1.6	2,739	0.7	1,411	2.2	4,233	0.9
55–59	1,092	2.6	3,276	1.2	1,548	2.8	4,644	1.4
60–64	804	4.8	2,412	2.3	1,258	5.1	3,774	3.0
65–69	709	8.0	2,127	5.2	960	9.4	2,880	6.3
70–74	679	13.4	2,037	14.0	1,070	17.3	3,210	16.2
75–79	498	26.2	1,494	21.3	974	28.2	2,922	30.4
80–84	322	40.1	966	31.9	676	43.8	2,028	42.9
85–89	152	54.2	456	43.4	344	59.4	1,032	47.9
90+	41	76.9	123	22.4	145	54.8	435	37.7

*Incidence per 1,000 person-years.

An univariate exploratory analysis of the presence of ADRD (regardless of the delay of onset) in the subgroup of chronic pain patients, taking into account the time of exposure to the different pain medications (according to molecules, therapeutic classes, and the DDDs), was performed to quantify the relationship between ADRD and medication exposure. This analysis was carried out only for exposures to each molecule/therapeutic class with a minimum of 400 individuals, as recommended for odds ratio calculations(Bewick et al., 2005).

The results are shown by frequency, associated percentage and odds ratio with their 95% confidence interval computed in univariate logistic regression (risk of ADRD according to exposure level). Statistics were computed using the SAS software. Tests were two-tailed and a *p* value <0.05 was considered statistically significant.

## Results

### Characteristics of study population

During the inclusion period (2006–2010), 15,136 individuals over 50 years old and with chronic pain were identified. After matching, 13,596 chronic pain and 40,788 control individuals were analyzed. As expected from the matching procedure, cases and controls were comparable in terms of age (66 years), sex (62% female), and Charlson score (0.4). Chronic pain and control individuals had a median follow-up time of 10.2 years. For the two groups and with similar proportions, the most common comorbidities were cancer, cerebrovascular disease, heart disease, and diabetes (Table 1). The vast majority of people with chronic pain were undergoing therapeutic management of their pain (94%), but the precise etiology of the chronic pain was not known in 82% of cases. Therapeutic pain management included non-opioid analgesics (~85%, mainly paracetamol), opioids (64%, mainly dextropropoxyphene), and anti-inflammatory drugs (topical and oral, 58 and 74% respectively; Table 3).

**Table 3:** Characteristics of chronic pain and pain medications.

	Chronic Pain group (*n* = 13,596)
Types of chronic pain (*n*, %)	
Neuropathic pain	538 (4.0)
Joint/rheumatic pain and arthropathy/spondylopathy	1,055 (7.8)
Back pain/Dorsopathy	944 (6.9)
Migraine/headache	4 (0.03)
Others pain	214 (1.6)
Unknown	11,197 (82.4)
Prescribed pain medications (*n*, %)	
Opioids	8,725 (64.2)
*Morphine*	835 (6.1)
*Opium*	0
*Oxycodone*	472 (3.5)
*Tramadol*	5,779 (42.5)
*Codeine*	3,478 (25.6)
*Fentanyl*	680 (5.0)
*Others*	5,079 (37.4)
Non-opioids analgesics (mainly paracetamol)	11,513 (84.7)
Triptans	695 (5.1)
Anti-inflammatory creams	7,814 (57.5)
Oral anti-inflammatory (NSAIDs)	10,030 (73.8)
Antiepileptics	2,062 (15.2)
*Gabapentin*	602 (4.4)
*Pregabalin*	1,758 (12.9)
Antidepressants	2,761 (20.3)
*TCAs*	1,248 (9.2)
*SNRIs*	1,915 (14.1)
Anesthetics (lidocaine)	2,720 (20.0)
No prescribed drug treatment	839 (6.2)

### Alzheimer’s disease and related dementia incidence

**Figure 1:**
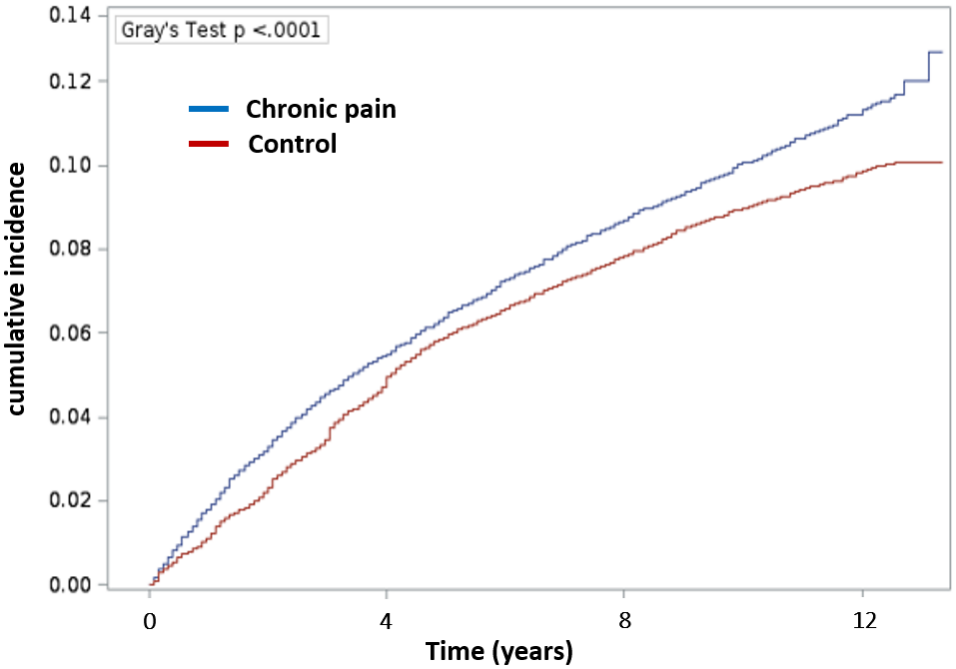
Cumulative incidence of Alzheimer’s disease and related dementias according to the presence of chronic pain. The incidence was calculated using the Fine and Gray method with lost of follow-up as censored and death as competitive risk. Blue: Chronic pain group and Red: Control group.

Survival analyzes with the Kaplan–Meier method showed that the death rate was greater (*p* < 0.001) in the chronic pain group [2,777 (20.4%)] than the control group [5,644 (13.8%)]. The same result was observed for the ADRD rate (and ADRD + deaths; Figure 1).

**Figure 2:**
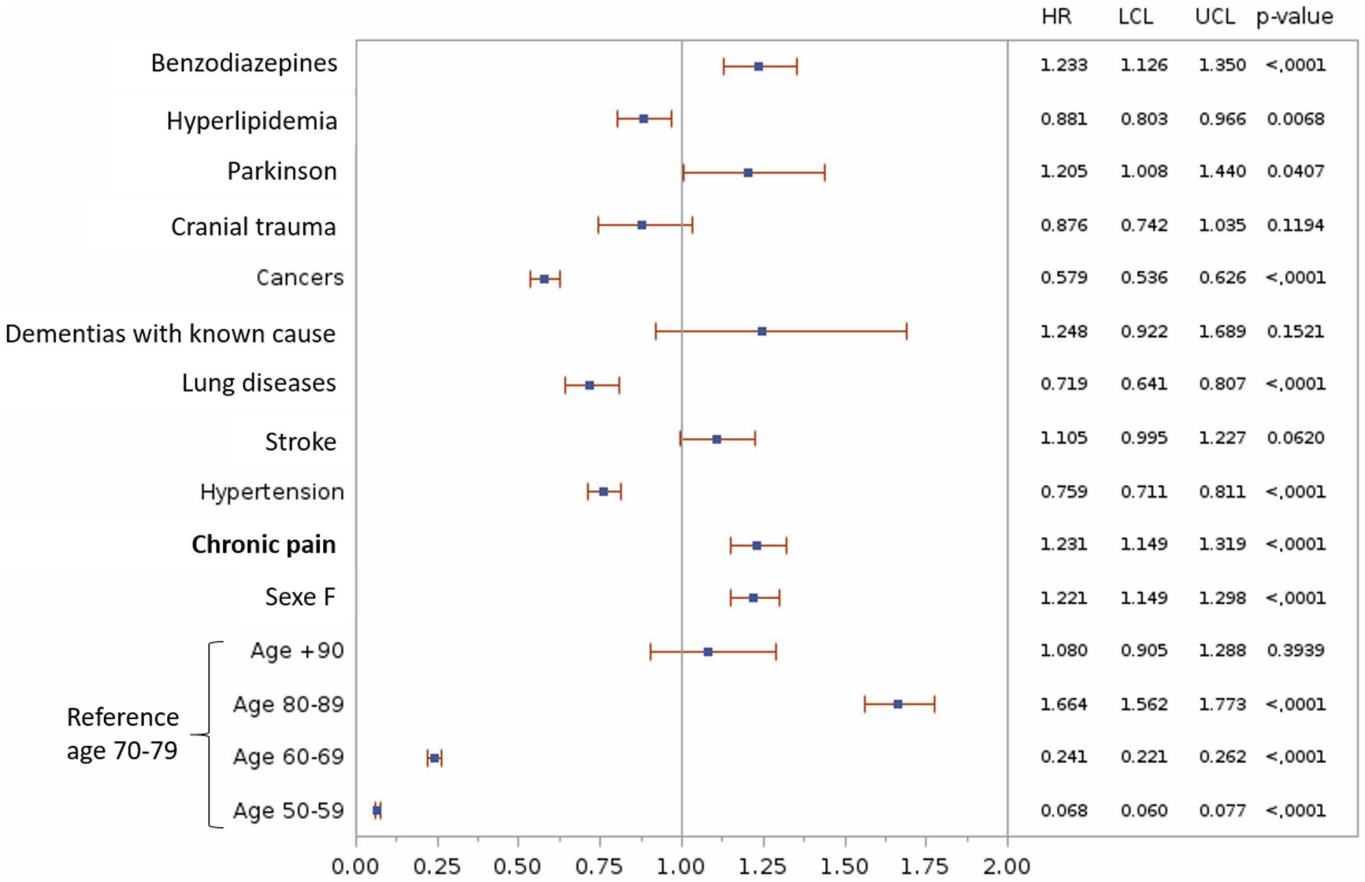
Multivariable analyze of the association between chronic pain and the risk of Alzheimer’s disease and related dementias. Only factors significantly related to the presence of ADRD in the univariate analysis are shown. HR, Hazard Ratio; LCL, Lower Confidence Limit; UCL, Upper Confidence Limit.

Therefore, Kaplan–Meier survival curves (univariate analysis), according to the Fine and Gray model with death as competing factor, were obtained (Figure 2). Among people with chronic pain, 1,416 (10.4%) developed ADRD with a median time to onset of 3.8 years (1.6–7.2), for a median age of onset of ADRD at 81 years, and an incidence rate of 11.3 cases per 1,000 patient-years. In control people, 3,692 (9.1%) developed dementia with a median time to onset of 4.1 years (2.1–6.8), with a median age at onset of ADRD at 82 years. The comparison of incidence between groups showed that control people had a lower incidence rate than people with chronic pain, with 9.5 cases per 1,000 patient-years (Gray’s test 18.67 and *p* < 0.0001). The analysis of ADRD onset in survival analysis, taking death as competing event, showed that people with chronic pain are at greater risk of ADRD onset (Gray’s test *p* < 0.0001).

Sensitivity analysis showed that the incidence of ADRD was higher for women (12.5 cases per 1,000 person-years vs. 9.2 cases for men) and increased significantly with age (50–59 year-olds, 1.6–2.2 cases per 1,000 person-years, +90 year-olds: 54.8–76.9 cases per 1,000 person-years, and according to sex; Table 2).

### Chronic pain as a risk factor for developing Alzheimer’s disease and related dementia

**Figure 3:**
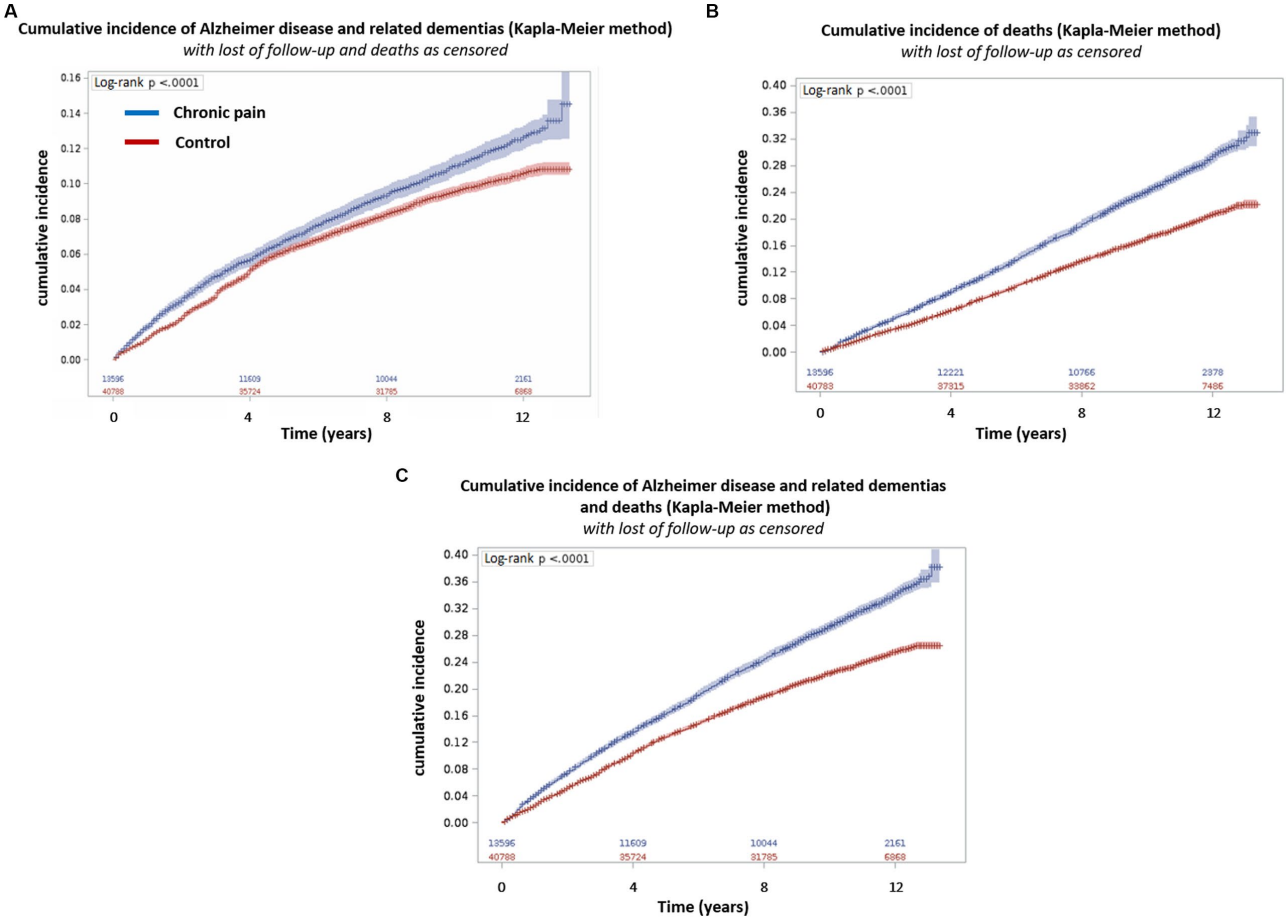
Cumulative incidence of Alzheimer’s disease and related dementias and/or death according to the presence of chronic pain. The incidence was calculated using the Kaplan Meier method. **(A)** Cumulative incidence of Alzheimer’s disease and related dementias with lost of follow-up and death as censored; **(B)** Cumulative incidence of deaths with lost of follow-up as censored; **(C)** Cumulative incidence of Alzheimer’s disease and related dementias and deaths with lost of follow-up as censored. Blue: Chronic pain group and Red: Control group.

Univariate analysis of confounding factors related to the development of ADRD showed that chronic pain increased the risk of developing ADRD [HR = 1.15 (1.08–1.22), *p* < 0.0001]. The same observation was made for confounders, such as Parkinson’s disease, dementia with known cause, stroke, benzodiazepine use, head injury, female sex and hypertension. In contrast, cancer, lung disease, and hyperlipidemia were protective against the development of ADRD (Figure 3). All of these confounders were therefore used for the adjusted model. Diabetes, depression, and epilepsy had no effect on the incidence of ADRD.

**Figure 4:**
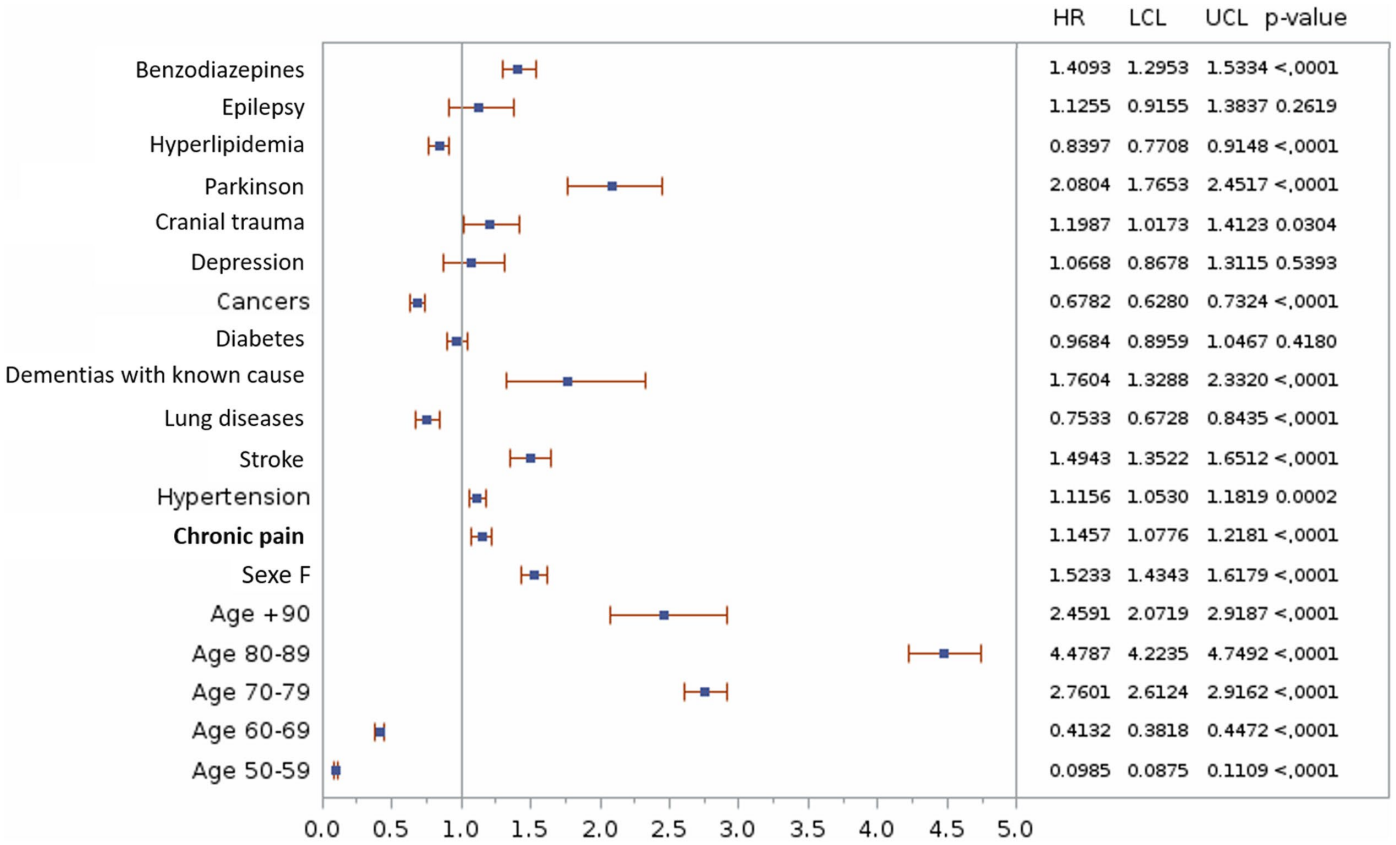
Univariate analyze of the association between chronic pain and the risk of Alzheimer’s disease and related dementias. HR, Hazard Ratio; LCL, Lower Confidence Limit; UCL, Upper Confidence Limit.

Multivariable analysis confirmed the previous results with an HR of 1.23 [1.15–1.32] of developing ADRD for the chronic pain group (Figure 4).

### Exploratory analyzes

Exploratory analyzes were performed to assess the relationship between exposure to classes of pain medications [NSAIDs, opioids, non-opioids (mainly paracetamol), antiepileptics, tricyclic antidepressants, serotonin and noradrenaline reuptake inhibitors, and anti-rheumatic] and the presence of ADRD. The analysis of DDDs showed that only the use of NSAIDs was correlated with a lower presence of ADRD and depended on the duration of exposure [mild exposure = OR 0.93, 95%CI (0.82–1.05); moderate exposure = OR 0.67, 95%CI (0.54–0.85); and strong exposure = OR 0.72, 95%CI (0.58–0.89)]. In contrast, only the use of non-opioid analgesics and opioids was correlated with a more frequent occurrence of ADRD, depending on the duration of exposure [non-opioid analgesics: mild exposure = OR 1.76, 95%CI (1.39–2.23); moderate exposure = OR 2.54, 95%CI (2.00–3.24); and strong exposure = OR 2.83, 95%CI (2.23–3.60); opioids: mild exposure = OR 1.10, 95%CI (0.97–1.24); moderate exposure = OR 1.21, 95%CI (1.00–1.47); and strong exposure = OR 1.24, 95%CI (1.02–1.49)]. Looking at all analgesic treatments, only continuous analgesic use over a 24-month period seems to be linked to the presence of ADRD [OR 1.23, 95%CI (1.10–1.38)] (Table 4). No relationship was found between the presence of ADRD and exposure to other therapeutic classes.

**Table 4:** Assessment of relationships between the therapeutic classes of pain medications exposures and development of Alzheimer’s disease and related dementias.

		ADRD		Odd Ratio OR [95%CI]	Value of *p*
		NO (*n* = 12,180)	YES (*n* = 1,416)		
NSAIDs–Defined daily dose DDD (months)					
0 DDD	*N*	3,842	493	REF	REF
	Frequency (%)	31,5	34,8		
<3 DDDs	*N*	6,064	720	0.93 [0.82–1.05]	0.210
	Frequency (%)	49,8	50,8		
≥3 and < = 12 DDDs**	*N*	1,123	97	**0.67 [0.54–0.85]**	**0.0007**
	Frequency (%)	9,2	6,9		
> 12 DDDs	*N*	1,151	106	**0.72 [0.58–0.89]**	**0.0031**
	Frequency (%)	9,4	7,5		
OPIOIDS–Defined daily dose DDD (months)					
0 DDD	*N*	5,098	549	REF	REF
	Frequency (%)	41,9	38,8		
<3 DDDs	*N*	4,713	551	1.10 [0.97–1.24]	0.153
	Frequency (%)	38,7	38,9		
≥3 and < = 15 DDDs	*N*	1,155	150	**1.21 [1.00–1.47]**	**0.050**
	Frequency (%)	9,5	10,6		
> 15 DDDs	*N*	1,214	166	**1.24 [1.02–1.49]**	**0.029**
	Frequency (%)	10,0	11,7		
NON-OPIOIDS–Defined daily dose DDD (months)					
0 DDD	*N*	1,535	86	REF	REF
	Frequency (%)	12,6	6,1		
<3 DDDs	*N*	5,245	518	**1.76 [1.39–2.23]**	**<0.0001**
	Frequency (%)	43,1	36,6		
≥3 and < = 18 DDDs	*N*	2,718	387	**2.54 [2.00–3.24]**	**<0.0001**
	Frequency (%)	22,3	27,3		
> 18 DDDs	*N*	2,682	425	**2.83 [2.23–3.60]**	**<0.0001**
	Frequency (%)	22,0	30,0		
ANTI-RHEUMATIC–Defined daily dose DDD (months)					
0 DDD	*N*	8,807	989	REF	REF
	Frequency (%)	72,3	69,8		
<3 DDDs	*N*	1,506	192	1.11 [0.94–1.30]	0.21
	Frequency (%)	12,4	13,6		
≥3 and < = 16 DDDs	*N*	920	118	1.11 [0.91–1.36]	0.32
	Frequency (%)	7,6	8,3		
> 16 DDDs	*N*	947	117	1.07 [0.88–1.31]	0.55
	Frequency (%)	7,8	8,3		
TRICYCLIC ANTIDEPRESSANTS–Defined daily dose DDD (months)					
0 DDD	*N*	11,060	1,288	REF	REF
	Frequency (%)	90,8	91,0		
<3 DDDs	*N*	918	107	1.02 [0.83–1.26]	0.91
	Frequency (%)	7,5	7,6		
≥3 and < = 15 DDDs	*N*	95	15	NA*	NA
	Frequency (%)	0,8	1,1		
> 15 DDDs	*N*	107	6	NA	NA
	Frequency (%)	0,9	0,4		
ANTIEPILEPTICS–Defined daily dose DDD (months)					
0 DDD	*N*	10,349	1,185	REF	REF
	Frequency (%)	85,0	83,7		
<3 DDDs	*N*	1,269	163	1.12 [0.94–1.33]	0.22
	Frequency (%)	10,4	11,5		
≥3 and < = 15 DDDs	*N*	275	38	NA	NA
	Frequency (%)	2,3	2,7		
> 15 DDDs	*N*	287	30	NA	NA
	Frequency (%)	2,4	2,1		
SNRI ANTIDEPRESSANTS–Defined daily dose DDD (months)					
0 DDD	*N*	12,031	1,403	REF	REF
	Frequency (%)	98,8	99,1		
<3 DDDs	*N*	52	9	NA	NA
	Frequency (%)	0,4	0,6		
≥3 and < = 22 DDDs	*N*	48	3	NA	NA
	Frequency (%)	0,4	0,2		
> 22 DDDs	*N*	49	1	NA	NA
	Frequency (%)	0,4	0,1		
ALL PAIN MEDICATIONS–Defined daily dose DDD (months)					
0 DDD	*N*	925	42	REF	REF
	Frequency (%)	7.6	3.0		
<3 DDDs	*N*	2,470	265	0.91 [0.79; 1.05]	0.17
	Frequency (%)	20.3	18.7		
≥3 and < = 24 DDDs	*N*	4,384	528	1.06 [0.95; 1.19]	0.35
	Frequency (%)	36.0	37.3		
> 24 DDDs	*N*	4,401	581	**1.23 [1.10; 1.38]**	**0.0003**
	Frequency (%)	36.1	41.0		

*NA; not applicable, minimum size not reached for odd ratio analysis. **Median of the distribution of DDDs in individuals with a DDD ≥ 3 months.

Bold value means statistically significant.

A second analysis was also carried out according to exposure to different analgesic molecules (see Supplementary material; Supplementary Table S1 for details). Nevertheless, the low rate of patients receiving certain drugs did not allow us to interpret reliably this results for all pain medications, only paracetamol and dextropropoxyphene widely prescribed were interpreted. We observed that paracetamol (OR from 1.75 to 2.80 according to exposure) and dextropropoxyphene (OR from 1.29 to 1.90 according to exposure) were correlated with the presence of ADRD and dependent on exposure time (Table 5). Nevertheless, these results are only exploratory and need to be confirmed by a dedicated study on more patients.

**Table 5:** Impact of the exposure of paracetamol and dextropropoxyphene on the presence of Alzheimer’s disease and related dementias.

Pain medications	Defined daily dose DDD (months)	Non-ADRD group *n* = 12,180 *n* (%)	ADRD group *n* = 1,416 *n* (%)	Odd Ratio OR [95%CI]	Value of *p*
Paracetamol	0 DDD	3,842 (31.5)	493 (34.8)	REF	REF
	<3 DDDs	6,064 (49.8)	720 (50.8)	1.75 [1.38–2.21]	**<0.0001**
	≥3 and < = 18 DDDs*	1,123 (9.2)	97 (6.8)	2.51 [1.97–3.19]	**<0.0001**
	> 18 DDDs	1,151 (9.4)	106 (7.5)	2.80 [2.20–3.55]	**<0.0001**
Dextropropoxyphene	0 DDD	1,535 (12.6)	86 (6.1)	REF	REF
	<3 DDDs	5,245 (43.1)	518 (36.6)	1.28 [1.14–1.45]	**<0.0001**
	≥3 and < = 12 DDDs**	2,718 (22.3)	387 (27.3)	1.45 [1.12–1.89]	**0.0051**
	> 12 DDDs	2,682 (22.0)	425 (30.0)	1.90 [1.49–2.41]	**<0.0001**

*Median of the distribution of DDDs in individuals with a DDD ≥ 3 months.

Bold value means statistically significant.

## Discussion

This retrospective study using administrative data examined the influence of chronic pain on the incidence of ADRD and provides recent data that are complementary to those in the literature. Our results showed that the presence of chronic pain is associated with a higher incidence and risk of developing ADRD when compared with older adults with no chronic pain. When exploring separately the different pain medications, a greater risk with long-term use of opioids and paracetamol was observed for developing ADRD. Although several cross-sectional/cohort studies reported association between chronic pain and ADRD (Whitlock et al., 2017; Khalid et al., 2020; Van der Leeuw et al., 2020; Kao et al., 2021; Rouch et al., 2021; Wang and Liu, 2021), only 2 studies assessed this relationship using national administrative health databases, but which not assess impact of pain medications. First one, the study by Kao et al. (2021) showed that patients with chronic pain had a higher risk of dementia than those without chronic pain [adjusted hazard ratio (AHR): 1.21; 95% confidence interval (CI): 1.15–1.26]. Second, the study by Khalid et al. (2020) showed that chronic pain was associated with incident ADRD [adjusted odds ratio (AOR) for any vs. no chronic pain = 1.21, 95% confidence interval (CI) = 1.04–1.40]. These results are similar to those in our study which confirms that chronic pain is a risk factor for the development of an ADRD.

Our results concerning the characteristics of people with chronic pain showed a higher comorbidity score than control at the end of the follow-up, consistent with the burden of chronic pain (Breivik et al., 2013; Cohen et al., 2021), and had similar characteristics in line with the few previous epidemiological studies assessing chronic pain (Bouhassira et al., 2008; Rapo-Pylkkö et al., 2016; Foley et al., 2021; Kerckhove et al., 2022). Similarly, ADRD individuals had characteristics found in the literature (Bauer et al., 2014; Eshetie et al., 2019). It should be noted that we find a higher mortality rate in individuals suffering from chronic pain, as previously demonstrated (Holmberg et al., 2020). Thus, in terms of external validity, our study populations can be considered representative of people with chronic pain or ADRD.

An important point concerns the confounding factors associated with chronic pain that impact cognition, such as depression, analgesic and psychotropic medications, and comorbidities. Previous literature has suggested that a number of factors are thought to influence the association between cognition and chronic pain, such as age, gender, depression, and benzodiazepine or opioid use (Armstrong, 2019). Our analysis took these different covariates into account, revealing that chronic pain *per se* was related to the development of ADRD, independent of these variables. Nevertheless, our study did not find certain risk factors for the development of ADRD previously described in the literature, such as diabetes (Kalaria, 2009; Ahtiluoto et al., 2010; Burillo et al., 2021) and depression (Byers and Yaffe, 2011). These differences in results may be explained by the different methodologies and populations used in the studies. In addition, the health care systems of the countries where these studies were conducted differ, with different prevention and care policies that may influence the weight of certain risk factors.

Although our study was not designed to demonstrate causality, many hypotheses have been advanced to explain potential mechanisms by which chronic pain may interfere with cognition. The characteristics of cognitive dysfunction associated with chronic pain have been described as a decrease in attention span and impaired memory function (Dick and Rashiq, 2007). The cognitive decline associated with chronic pain is consistent with brain imaging findings that reveal an overlap between frontal regions involved in pain modulation and cognitive function (Bushnell et al., 2013), and more broadly with the pain matrix, with a possible reversible effect after pain treatment (Seminowicz et al., 2011). In the literature, chronic pain has been shown to promote detrimental neurostructural and neurofunctional changes. For example, neuroimaging has demonstrated neurodegenerative changes in chronic pain subjects identical to those observed in ADRD (Cao et al., 2019), including a reduction in gray matter volume in the amygdala, hippocampus, and frontal cortices, brain regions integrally involved in cognitive and behavioral functioning (Malfliet et al., 2017). Similarly, chronic pain may contribute to these neurodegenerative changes by promoting neuroinflammation (Calsolaro and Edison, 2016), such as microglial, also involved in the pathogenesis of Alzheimer’s disease through the production of beta-amyloid plaques and neurofibrillary tangles (Cao et al., 2019). Neuroinflammation also affects neuroplasticity and synaptic performance through the reduction of brain-derived neurotrophic factors (Malfliet et al., 2017).

Finally, concerning pain medications exposure, NSAIDs have been shown to be correlated with a lower presence of ADRD. In contrast, non-opioid analgesics (mainly paracetamol) and opioids were associated with a greater presence of ADRD. These results are consistent with those previously published, showing a protective effect of NSAIDs against dementia (Szekely et al., 2008). Likewise for paracetamol (Jones, 2014) and opioids (Dublin et al., 2015), which were shown to be risk factors for the development of dementia. Unfortunately, our results are only exploratory, the low prescription rate of several drugs did not allow us to assess precisely and reliably the correlation with the presence of ADRD for each pain medication. The existing literature demonstrates that there are other risks associated with opioid use in the elderly, but the data available to date do not suggest that opioid use causes substantial long-term cognitive damage. Regarding paracetamol, our results should be taken with caution. To date, there is no reliable study showing a link between paracetamol consumption and the development of cognitive disorders (Jones, 2014). Moreover, the difficulty in separating the effect of pain from the effect of its treatment makes the interpretation of such exploratory results even more complicated. A specific study on a larger number of patients and using an adequate methodology is necessary to answer these issues.

### Limitations

First, the identification of people suffering from chronic pain can only be done by medical diagnosis, LTI or the presence of a continuity of pain medications. This implies that individuals who have not been diagnosed for their chronic pain and who are not treated by reimbursed painkillers are not identified, with a potential underestimation of chronic pain patients. Second limitation is the reliability of the data reported in the administrative databases, with risks of error or non-coding of some pathologies or the care received. This is the case for chronic pain (Lacasse et al., 2020) and ADRD (Carcaillon-Bentata et al., 2016), leading to the potential underestimation of theses pathologies. Nevertheless, the algorithms used to identify chronic pain and ADRD individuals seemed reliable. Indeed, in a previous study (Chenaf et al., 2018) the algorithm used for identifying chronic pain showed a prevalence of chronic pain in France similar to that found in a large French national cohort study (Bouhassira et al., 2008), indirectly validating the algorithm. Concerning the algorithm of identification of ADRD, it has been validated and published previously (Rochoy et al., 2019a; Elyn et al., 2022). Finally, the most important limitation was the time between the identification of chronic pain (index date) and the diagnosis of ADRD. In a similar study of Billioti de Gage et al. (2014) the minimal delay used before dementia onset was 5 years. In our study, the average time from identification of chronic pain to diagnosis of ADRD was approximately 4 years. However, because of a lack of power (not enough patients with a minimum of 5 years between chronic pain and ADRD), we did not impose a delay between the index date and the diagnosis of ADRD. This lack of patients is explained by the advanced age of our population at inclusion (65 years), leaving little time for the onset of ADRD. Moreover, as the EGB database only had data available from 2005 onwards, the follow-up time was too limited to include younger patients and to allow the appearance of ADRD. This limitation raises the question of follow-up bias. Indeed, it is possible that individuals with chronic pain have a more optimal medical follow-up than individuals without chronic pain, and indirectly have an earlier detection of dementia disorders and therefore an earlier diagnosis. However, this follow-up bias needs to be modulated, as control individuals, like those with chronic pain, have similar comorbidities, some of which require extensive medical follow-up. These limitations raises questions about the real impact of chronic pain on the incidence of ADRD. Moreover, certain mechanisms involved in pathological central nervous system aging toward ADRD start very early even before the mild cognitive impairment stages (Jack et al., 2010; Bateman et al., 2012). Moreover, the delay in diagnosis is estimated to be between 2 and 5 years after the onset of symptoms, with more than two-thirds of diagnoses already in moderate to severe stages (Löppönen et al., 2003). Thus, we can hypothesize that chronic pain could be considered as a risk factor, but not the direct cause, of a neurodegenerative pathology and its clinical revelation. Also, symptoms of chronic pain may be a prodromal of ADRD. These questions remain complex and difficult to answer. A larger study, including a minimum delay of 5 years between chronic pain and the diagnosis of ADRD, should be conducted to confirm our results.

In conclusion, our results suggest that chronic pain is a risk factor for the development of an ADRD independently of various confounders. Combined with the fact that chronic pain can lead to decreased mobility, interfere with daily activities, and increase the risk of falls and additional injuries, our study reinforces the importance of prevention, diagnosis, and management of chronic pain with adapted pharmacological and non-pharmacological strategies to limit resulting comorbidities, such as dementias and Alzheimer’s disease. This is especially important in the elderly, who may have difficulty expressing their pain and are at greater risk of developing dementias. Nevertheless, further studies are needed to clarify the pathophysiological mechanisms involved in relationship between chronic pain, long-term use of pain medications and dementia (Cao et al., 2019).

## Data availability statement

The datasets presented in this article are not readily available because French law prohibits the authors from directly sharing the data used for this study, but access can be requested directly from SNDS (website: https://www.health-data-hub.fr).

## Ethics statement

The studies involving humans were approved by IRB00013412, “CHU de Clermont-Ferrand IRB #1,” IRB number 2022-CF039. The studies were conducted in accordance with the local legislation and institutional requirements. Written informed consent for participation was not required from the participants or the participants’ legal guardians/next of kin in accordance with the national legislation and institutional requirements.

## Author contributions

NK, NB, AM, and CB contributed to conception and design of the study. NB and AM organized the database and performed the statistical analysis. NB wrote the first draft of the manuscript. NK, AM, and NB wrote sections of the manuscript. All authors contributed to the article and approved the submitted version.
