# Supplementary material for: Chronic pain is a risk factor for incident Alzheimer’s disease: a nationwide propensity-matched cohort using administrative data
Source: Front Aging Neurosci. 2023 Sep 28;15:1193108. doi: 10.3389/fnagi.2023.1193108 (PMC10575742; doi:10.3389/fnagi.2023.1193108)
Supplement: Supplementary file 1 [file Data_Sheet_1.docx]

*Matching*

The 3:1 matching was performed on propensity score (age, sex, and Charlson Comorbidities Index – CCI score) with a caliper ±0.005. Comorbidities were grouped using the CCI score per individual. The comorbidities analyzed and their associated ICD-10 codes are as follows:

Myocardial Infarction (I21', 'I22','I252); Congestive Heart Failure ('I43', 'I50', 'I099', 'I110', 'I130', 'I132', 'I255', 'I420', 'I425', 'I426', 'I427', 'I428', 'I429', 'P290'); Peripheral Vascular Disease ('I70', 'I71', 'I731', 'I738', 'I739', 'I771', 'I790', 'I792', 'K551', 'K558', 'K559', 'Z958', 'Z959'); Cerebrovascular Disease ('G45','G46','I60','I61','I62','I63','I64','I65','I66','I67','I68','I69','H340'); Dementia ('F00', 'F01', 'F02', 'F03', 'G30', 'F051', 'G311'); Chronic Pulmonary Disease ('J40', 'J41', 'J42', 'J43', 'J44', 'J45', 'J46', 'J47', 'J60', 'J61', 'J62', 'J63', 'J64', 'J65', 'J66', 'J67','I278', 'I279', 'J684', 'J701', 'J703'); Connective Tissue Disease-Rheumatic Disease ('M05', 'M32', 'M33', 'M34', 'M06', 'M315', 'M351', 'M353', 'M360'); Peptic Ulcer Disease ('K25', 'K26', 'K27', 'K28'); Mild Liver Disease ('B18', 'K73', 'K74', 'K700', 'K701', 'K702', 'K703', 'K709', 'K717', 'K713', 'K714', 'K715', 'K760', 'K762', 'K763', 'K764', 'K768', 'K769', 'Z944'); Diabetes without complications ('E100', 'E101', 'E106', 'E108', 'E109', 'E110', 'E111', 'E116', 'E118', 'E119', 'E120', 'E121', 'E126', 'E128', 'E129', 'E130', 'E131', 'E136', 'E138', 'E139', 'E140', 'E141', 'E146', 'E148', 'E149'); Diabetes with complications ('E102', 'E103', 'E104', 'E105', 'E107', 'E112', 'E113', 'E114', 'E115', 'E117', 'E122', 'E123', 'E124', 'E125', 'E127', 'E132', 'E133', 'E134', 'E135', 'E137', 'E142', 'E143', 'E144', 'E145', 'E147'); Paraplegia and Hemiplegia ('G81', 'G82', 'G041', 'G114', 'G801', 'G802', 'G830', 'G831', 'G832', 'G833', 'G834', 'G839'); Renal Disease ('N18', 'N19', 'N052', 'N053', 'N054', 'N055', 'N056', 'N057', 'N250', 'I120', 'I131', 'N032', 'N033', 'N034', 'N035', 'N036', 'N037', 'Z490', 'Z491', 'Z492', 'Z940', 'Z992'); Cancer ('C00', 'C01', 'C02', 'C03', 'C04', 'C05', 'C06', 'C07', 'C08', 'C09', 'C10', 'C11', 'C12', 'C13', 'C14', 'C15', 'C16', 'C17', 'C18', 'C19', 'C20', 'C21', 'C22', 'C23', 'C24', 'C25', 'C26', 'C30', 'C31', 'C32', 'C33', 'C34', 'C37', 'C38', 'C39', 'C40', 'C41', 'C43', 'C45', 'C46', 'C47', 'C48', 'C49', 'C50', 'C51', 'C52', 'C53', 'C54', 'C55', 'C56', 'C57', 'C58', 'C60', 'C61', 'C62', 'C63', 'C64', 'C65', 'C66', 'C67', 'C68', 'C69', 'C70', 'C71', 'C72', 'C73', 'C74', 'C75', 'C76', 'C81', 'C82', 'C83', 'C84', 'C85', 'C88', 'C90', 'C91', 'C92', 'C93', 'C94', 'C95', 'C96', 'C97'); Moderate or Severe Liver Disease ('K704', 'K711', 'K721', 'K729', 'K765', 'K766', 'K767', 'I850', 'I859', 'I864', 'I982'); Metastatic Carcinoma ('C77', 'C78', 'C79', 'C80'); AIDS/HIV ('B20', 'B21', 'B22', 'B24').

*Study sample: selection of patients with ADRD*

According to previous publications, the ICD-10 codes used to identify ADRD were for diagnosis: F00 (Alzheimer's disease related dementia), F01 (Vascular dementia), F03 (Dementia, unspecified), G30 (Alzheimer’s disease), G310 (Circumscribed cerebral atrophy), G311 (Senile cerebral degeneration, not elsewhere classified), and G319 (Degenerative disease of the nervous system, unspecified), and for treatments: the ATC codes N06DA (anticholinesterase) and N06DX01 (memantine). To avoid erroneous inclusion of individuals, individuals had to have at least two dates of diagnosis of ADRD, or two dispensations of cognitive stimulants, or to have a diagnosis of ADRD associated with a dispensation of cognitive stimulants.

*Study sample: selection of patients with chronic pain*

According to previous publications, only chronic pain as a principal, related, or associated diagnosis with the reason for hospitalization or long-term illness was identified by ICD-10 codes R521 (intractable neuropathic pain), R522 (other chronic pain), and M797 (fibromyalgia). We have restricted our selection to these codes because they are the only reliable ones to confirm the presence of chronic pain. Other codes that could indicate acute pain, such as M00 to M99 (musculoskeletal pain) or R529 (unspecified pain), were not selected.

All analgesic drugs (ATC code: N02), oral anti-inflammatory and antirheumatic drugs (M01), and topical anti-inflammatory drugs (M02A) were searched. First and second line drugs for neuropathic pain were also searched: antiepileptics (gabapentin N03AX12 and pregabalin N03AX16), tricyclic antidepressants (clomipramine N06AA04, amitriptyline N06AA09 and nortriptyline N06AA10), serotonin-norepinephrine reuptake inhibitors (venlafaxine N06AX16 and duloxetine N06AX21) and lidocaine plasters (N01BB02 and N01BB52). The presence of CP was confirmed only in case of continuous and ongoing treatment during the inclusion period, for at least 6 consecutive months with an interval of less than 35 days between two consecutive prescriptions. The interval of 35 days is based on the fact that in France, prescribed drugs are delivered for a maximum duration of 4 weeks, to which a grace period of 7 days is added to avoid wrongly concluding that there is a break in continuity of treatment. This methodology prevented us from including patients with probably non-chronic pain.

*Details of pain medications analyzed for exploratory analysis according to their therapeutic classes*

**Opioids** = CODEINE, FENTANYL, HYDROMORPHONE, MORPHINE, OPIUM, OXYCODONE, TRAMADOL and DEXTROPOXYPHENE

**Non-opioids** = PARACETAMOL and NEFOPAM (Paracetamol accounts for +90% of cases).

**SNRI antidepressants** = DULOXETINE and VENLAFAXINE

**TCAs antidepressants** = AMITRIPTYLINE and CLOMIPRAMINE

**Antiepileptics** = GABAPENTIN and PREGABALIN

**Anti-rheumatic** = CHONDROITINE, DIACEREINE, GLUCOSAMINE and NIFLUMIC ACID

**NSAIDs** = ACECLOFENAC, ASPIRIN, ALMINOPROFEN, CELECOXIB, DICLOFENAC, ETODOLAC, ETORICOXIB, FENOPROFEN, FLOCTAFENINE, FLURBIPROFEN, IBUPROFEN, KETOPROFEN, MEFENAMIC ACID, MELOXICAM, MORNIFLUMATE, NABUMETONE, NAPROXEN, NIMESULIDE, PIROXICAM, SULINDAC, TENOXICAM and TIAPROFENIC ACID

**Triptans and Anesthetics** classes were not included due to lack of individuals with prescription.
