# Supplementary material for: Chronic pain is a risk factor for incident Alzheimer’s disease: a nationwide propensity-matched cohort using administrative data
Source: Front Aging Neurosci. 2023 Sep 28;15:1193108. doi: 10.3389/fnagi.2023.1193108 (PMC10575742; doi:10.3389/fnagi.2023.1193108)
Supplement: Supplementary file 2 [file Table_1.docx]

**Supplementary Table 1. Assessment of relationships between pain medications exposures and development of Alzheimer’s disease and related dementias**

|  |  | **ADRD** | | **Odd Ratio  OR [95%CI]** | **p-value** |
| --- | --- | --- | --- | --- | --- |
|  |  | **NO**  **(n=12180)** | **YES**  **(n=1416)** |  |  |
| **ACECLOFENAC** | | | | | |
| 0 DDD* | N | 11362 | 1318 | REF | REF |
|  | Frequency (%) | 93,3 | 93,1 |  |  |
| <180 DDDs | N | 746 | 92 | 1.07 [0.86;1.34] | .62 |
|  | Frequency (%) | 6,1 | 6,5 |  |  |
| ≥180 and <= 315 DDDs | N | 37 | 3 | NA** | NA |
|  | Frequency (%) | 0,3 | 0,2 |  |  |
| > 315 DDDs | N | 35 | 3 | NA | NA |
|  | Frequency (%) | 0,3 | 0,2 |  |  |
| **ASPIRIN** | | | | | |
| 0 DDD | N | 10994 | 1256 | REF | REF |
|  | Frequency (%) | 90,3 | 88,7 |  |  |
| <180 DDDs | N | 1134 | 157 | **1.21 [1.01;1.44]** | **.035** |
|  | Frequency (%) | 9,3 | 11,1 |  |  |
| ≥180 and <= 233 DDDs | N | 25 | 2 | NA | NA |
|  | Frequency (%) | 0,2 | 0,1 |  |  |
| > 233 DDDs | N | 27 | 1 | NA | NA |
|  | Frequency (%) | 0,2 | 0,1 |  |  |
| **AMITRIPTYLINE** | | | | | |
| 0 DDD | N | 11260 | 1307 | REF | REF |
|  | Frequency (%) | 92,4 | 92,3 |  |  |
| <180 DDDs | N | 815 | 100 | 1.06 [0.85;1.31] | .64 |
|  | Frequency (%) | 6,7 | 7,1 |  |  |
| ≥180 and <= 487 DDDs | N | 51 | 6 | NA | NA |
|  | Frequency (%) | 0,4 | 0,4 |  |  |
| > 487 DDDs | N | 54 | 3 | NA | NA |
|  | Frequency (%) | 0,4 | 0,2 |  |  |
| **CARBASALATE** | | | | | |
| 0 DDD | N | 12178 | 1416 | REF | REF |
|  | Frequency (%) | 100,0 | 100,0 |  |  |
| <180 DDDs | N | 2 | 0 | NA | NA |
|  | Frequency (%) | 0,0 | 0,0 |  |  |
| ≥180 | N | 0 | 0 | NA | NA |
|  | Frequency (%) | 0,0 | 0,0 |  |  |
| **CELECOXIB** | | | | | |
| 0 DDD | N | 11000 | 1273 | REF | REF |
|  | Frequency (%) | 90,3 | 89,9 |  |  |
| <180 DDDs | N | 945 | 126 | 1.16 [0.95;1.41] | .15 |
|  | Frequency (%) | 7,8 | 8,9 |  |  |
| ≥180 and <= 330 DDDs | N | 125 | 10 | NA | NA |
|  | Frequency (%) | 1,0 | 0,7 |  |  |
| > 330 DDDs | N | 110 | 7 | NA | NA |
|  | Frequency (%) | 0,9 | 0,5 |  |  |
| **CLOMIPRAMINE** | | | | | |
| 0 DDD | N | 11927 | 1390 | REF | REF |
|  | Frequency (%) | 97,9 | 98,2 |  |  |
| <180 DDDs | N | 149 | 14 | NA | NA |
|  | Frequency (%) | 1,2 | 1,0 |  |  |
| ≥180 and <= 409 DDDs | N | 49 | 9 | NA | NA |
|  | Frequency (%) | 0,4 | 0,6 |  |  |
| > 409 DDDs | N | 55 | 3 | NA | NA |
|  | Frequency (%) | 0,5 | 0,2 |  |  |
| **DEXTROPROPOXYPHENE** | | | | | |
| 0 DDD | N | 1535 | 86 | REF | REF |
|  | Frequency (%) | 12,6 | 6,1 |  |  |
| <180 DDDs | N | 5245 | 518 | **1.28 [1.14-1.45]** | **<.0001** |
|  | Frequency (%) | 43,1 | 36,6 |  |  |
| ≥180 and <= 365 DDDs | N | 2718 | 387 | **1.45 [1.12-1.89]** | **0.0051** |
|  | Frequency (%) | 22,3 | 27,3 |  |  |
| > 365 DDDs | N | 2682 | 425 | **1.90 [1.49-2.41]** | **<.0001** |
|  | Frequency (%) | 22,0 | 30,0 |  |  |
| **DIACEREINE** | | | | | |
| 0 DDD | N | 11049 | 1271 | REF | REF |
|  | Frequency (%) | 90,7 | 89,8 |  |  |
| <180 DDDs | N | 587 | 82 | 1.21 [0.95;1.54] | .12 |
|  | Frequency (%) | 4,8 | 5,8 |  |  |
| ≥180 and <= 390 DDDs | N | 275 | 37 | NA | NA |
|  | Frequency (%) | 2,3 | 2,6 |  |  |
| > 365 DDDs | N | 269 | 26 | NA | NA |
|  | Frequency (%) | 2,2 | 1,8 |  |  |
| **DICLOFENAC** | | | | | |
| 0 DDD | N | 9002 | 1073 | REF | REF |
|  | Frequency (%) | 73,9 | 75,8 |  |  |
| <180 DDDs | N | 2670 | 286 | 0.90 [0.79;1.03] | .15 |
|  | Frequency (%) | 21,9 | 20,2 |  |  |
| ≥180 | N | 508 | 57 | 0.96 [0.73;1.27] | .85 |
|  | Frequency (%) | 4,2 | 4,0 |  |  |
| **DULOXETINE** | | | | | |
| 0 DDD | N | 11579 | 1338 | REF | REF |
|  | Frequency (%) | 95,1 | 94,5 |  |  |
| <180 DDDs | N | 256 | 34 | NA | NA |
|  | Frequency (%) | 2,1 | 2,4 |  |  |
| ≥180 and <= 672 DDDs | N | 168 | 27 | NA | NA |
|  | Frequency (%) | 1,4 | 1,9 |  |  |
| > 672 DDDs | N | 177 | 17 | NA | NA |
|  | Frequency (%) | 1,5 | 1,2 |  |  |
| **CODEINE** | | | | | |
| 0 DDD | N | 9035 | 1077 | REF | REF |
|  | Frequency (%) | 74,2 | 76,1 |  |  |
| <180 DDDs | N | 2541 | 274 | 0.91 [0.79;1.05] | .20 |
|  | Frequency (%) | 20,9 | 19,4 |  |  |
| ≥180 and <= 432 DDDs | N | 298 | 38 | NA | NA |
|  | Frequency (%) | 2,4 | 2,7 |  |  |
| > 432 DDDs | N | 306 | 27 | NA | NA |
|  | Frequency (%) | 2,5 | 1,9 |  |  |
| **ERGOTAMINE** | | | | | |
| 0 DDD | N | 11927 | 1389 | REF | REF |
|  | Frequency (%) | 97,9 | 98,1 |  |  |
| <180 DDDs | N | 111 | 11 | NA | NA |
|  | Frequency (%) | 0,9 | 0,8 |  |  |
| ≥180 and <= 360 DDDs | N | 17 | 5 | NA | NA |
|  | Frequency (%) | 0,1 | 0,4 |  |  |
| > 360 DDDs | N | 125 | 11 | NA | NA |
|  | Frequency (%) | 1,0 | 0,8 |  |  |
| **ELETRIPTAN** | | | | | |
| 0 DDD | N | 12109 | 1411 | REF | REF |
|  | Frequency (%) | 99,4 | 99,6 |  |  |
| <180 DDDs | N | 57 | 5 | NA | NA |
|  | Frequency (%) | 0,5 | 0,4 |  |  |
| ≥180 and <= 305 DDDs | N | 7 | 0 | NA | NA |
|  | Frequency (%) | 0,1 | 0,0 |  |  |
| > 305 DDDs | N | 7 | 0 | NA | NA |
|  | Frequency (%) | 0,1 | 0,0 |  |  |
| **ETODOLAC** | | | | | |
| 0 DDD | N | 11957 | 1389 | REF | REF |
|  | Frequency (%) | 98,2 | 98,1 |  |  |
| <180 DDDs | N | 216 | 25 | NA | NA |
|  | Frequency (%) | 1,8 | 1,8 |  |  |
| ≥180 and <= 230 DDDs | N | 4 | 1 | NA | NA |
|  | Frequency (%) | 0,0 | 0,1 |  |  |
| > 230 DDDs | N | 3 | 1 | NA | NA |
|  | Frequency (%) | 0,0 | 0,1 |  |  |
| **ETORICOXIB** | | | | | |
| 0 DDD | N | 11874 | 1375 | REF | REF |
|  | Frequency (%) | 97,5 | 97,1 |  |  |
| <180 DDDs | N | 234 | 30 | NA | NA |
|  | Frequency (%) | 1,9 | 2,1 |  |  |
| ≥180 and <= 364 DDDs | N | 37 | 5 | NA | NA |
|  | Frequency (%) | 0,3 | 0,4 |  |  |
| > 364 DDDs | N | 35 | 6 | NA | NA |
|  | Frequency (%) | 0,3 | 0,4 |  |  |
| **FENOPROFEN** | | | | | |
| 0 DDD | N | 12141 | 1409 | REF | REF |
|  | Frequency (%) | 99,7 | 99,5 |  |  |
| <180 DDDs | N | 37 | 7 | NA | NA |
|  | Frequency (%) | 0,3 | 0,5 |  |  |
| ≥180 and <= 706 DDDs | N | 1 | 0 | NA | NA |
|  | Frequency (%) | 0,0 | 0,0 |  |  |
| > 706 DDDs | N | 1 | 0 | NA | NA |
|  | Frequency (%) | 0,0 | 0,0 |  |  |
| **FENTANYL** | | | | | |
| 0 DDD | N | 11684 | 1330 | REF | REF |
|  | Frequency (%) | 95,9 | 93,9 |  |  |
| <180 DDDs | N | 269 | 62 | NA | NA |
|  | Frequency (%) | 2,2 | 4,4 |  |  |
| ≥180 | N | 227 | 24 | NA | NA |
|  | Frequency (%) | 1,9 | 1,7 |  |  |
| **FLOCTAFENINE** | | | | | |
| 0 DDD | N | 12114 | 1400 | REF | REF |
|  | Frequency (%) | 99,5 | 98,9 |  |  |
| <180 DDDs | N | 62 | 15 | NA | NA |
|  | Frequency (%) | 0,5 | 1,1 |  |  |
| ≥180 and <= 370 DDDs | N | 3 | 0 | NA | NA |
|  | Frequency (%) | 0,0 | 0,0 |  |  |
| > 370 DDDs | N | 1 | 1 | NA | NA |
|  | Frequency (%) | 0,0 | 0,1 |  |  |
| **FLURBIPROFEN** | | | | | |
| 0 DDD | N | 11785 | 1375 | REF | REF |
|  | Frequency (%) | 96,8 | 97,1 |  |  |
| <180 DDDs | N | 306 | 29 | NA | NA |
|  | Frequency (%) | 2,5 | 2,0 |  |  |
| ≥180 and <= 439 DDDs | N | 49 | 6 | NA | NA |
|  | Frequency (%) | 0,4 | 0,4 |  |  |
| > 439 DDDs | N | 40 | 6 | NA | NA |
|  | Frequency (%) | 0,3 | 0,4 |  |  |
| **GABAPENTIN** | | | | | |
| 0 DDD | N | 11661 | 1333 | REF | REF |
|  | Frequency (%) | 95,7 | 94,1 |  |  |
| <180 DDDs | N | 370 | 69 | **1.64 [1.26;2.13]** | **<.0001** |
|  | Frequency (%) | 3,0 | 4,9 |  |  |
| ≥180 and <= 426 DDDs | N | 75 | 8 | NA | NA |
|  | Frequency (%) | 0,6 | 0,6 |  |  |
| > 426 DDDs | N | 74 | 6 | NA | NA |
|  | Frequency (%) | 0,6 | 0,4 |  |  |
| **GLUCOSAMINE** | | | | | |
| 0 DDD | N | 11727 | 1353 | REF | REF |
|  | Frequency (%) | 96,3 | 95,6 |  |  |
| <180 DDDs | N | 285 | 41 | NA | NA |
|  | Frequency (%) | 2,3 | 2,9 |  |  |
| ≥180 and <= 378 DDDs | N | 88 | 10 | NA | NA |
|  | Frequency (%) | 0,7 | 0,7 |  |  |
| > 378 DDDs | N | 80 | 12 | NA | NA |
|  | Frequency (%) | 0,7 | 0,8 |  |  |
| **HYDROMORPHONE** | | | | | |
| 0 DDD | N | 12155 | 1415 | REF | REF |
|  | Frequency (%) | 99,8 | 99,9 |  |  |
| <180 DDDs | N | 10 | 0 | NA | NA |
|  | Frequency (%) | 0,1 | 0,0 |  |  |
| ≥180 and <= 672 DDDs | N | 8 | 0 | NA | NA |
|  | Frequency (%) | 0,1 | 0,0 |  |  |
| > 672 DDDs | N | 7 | 1 | NA | NA |
|  | Frequency (%) | 0,1 | 0,1 |  |  |
| **IBUPROFEN** | | | | | |
| 0 DDD | N | 8805 | 1097 | REF | REF |
|  | Frequency (%) | 72,3 | 77,5 |  |  |
| <180 DDDs | N | 3159 | 300 | **0.77 [0.67;0.88]** | **.0001** |
|  | Frequency (%) | 25,9 | 21,2 |  |  |
| ≥180 and <= 304 DDDs | N | 109 | 10 | NA | NA |
|  | Frequency (%) | 0,9 | 0,7 |  |  |
| > 304 DDDs | N | 107 | 9 | NA | NA |
|  | Frequency (%) | 0,9 | 0,6 |  |  |
| **KETOPROFEN** | | | | | |
| 0 DDD | N | 8836 | 1114 | REF | REF |
|  | Frequency (%) | 72,5 | 78,7 |  |  |
| <180 DDDs | N | 3039 | 283 | **0.75 [0.65;0.86]** | **<.0001** |
|  | Frequency (%) | 25,0 | 20,0 |  |  |
| ≥180 and <= 330 DDDs | N | 157 | 9 | NA | NA |
|  | Frequency (%) | 1,3 | 0,6 |  |  |
| > 330 DDDs | N | 148 | 10 | NA | NA |
|  | Frequency (%) | 1,2 | 0,7 |  |  |
| **MELOXICAM** | | | | | |
| 0 DDD | N | 11825 | 1375 | REF | REF |
|  | Frequency (%) | 97,1 | 97,1 |  |  |
| <180 DDDs | N | 326 | 39 | NA | NA |
|  | Frequency (%) | 2,7 | 2,8 |  |  |
| ≥180 and <= 336 DDDs | N | 14 | 2 | NA | NA |
|  | Frequency (%) | 0,1 | 0,1 |  |  |
| > 336 DDDs | N | 15 | 0 | NA | NA |
|  | Frequency (%) | 0,1 | 0,0 |  |  |
| **MORNIFLUMATE** | | | | | |
| 0 DDD | N | 12134 | 1411 | REF | REF |
|  | Frequency (%) | 99,6 | 99,6 |  |  |
| <180 DDDs | N | 44 | 5 | NA | NA |
|  | Frequency (%) | 0,4 | 0,4 |  |  |
| ≥180 and <= 702 DDDs | N | 1 | 0 | NA | NA |
|  | Frequency (%) | 0,0 | 0,0 |  |  |
| > 702 DDDs | N | 1 | 0 | NA | NA |
|  | Frequency (%) | 0,0 | 0,0 |  |  |
| **MORPHINE** | | | | | |
| 0 DDD | N | 11490 | 1329 | REF | REF |
|  | Frequency (%) | 94,3 | 93,9 |  |  |
| <180 DDDs | N | 548 | 78 | 1.24 [0.97;1.58] | .099 |
|  | Frequency (%) | 4,5 | 5,5 |  |  |
| ≥180 and <= 453 DDDs | N | 71 | 5 | NA | NA |
|  | Frequency (%) | 0,6 | 0,4 |  |  |
| > 453 DDDs | N | 71 | 4 | NA | NA |
|  | Frequency (%) | 0,6 | 0,3 |  |  |
| **NABUMETONE** | | | | | |
| 0 DDD | N | 11519 | 1333 | REF | REF |
|  | Frequency (%) | 94,6 | 94,1 |  |  |
| <180 DDDs | N | 658 | 82 | 1.08 [0.85;1.37] | .58 |
|  | Frequency (%) | 5,4 | 5,8 |  |  |
| ≥180 and <= 336 DDDs | N | 2 | 0 | NA | NA |
|  | Frequency (%) | 0,0 | 0,0 |  |  |
| > 336 DDDs | N | 1 | 1 | NA | NA |
|  | Frequency (%) | 0,0 | 0,1 |  |  |
| **NAPROXEN** | | | | | |
| 0 DDD | N | 10645 | 1303 | REF | REF |
|  | Frequency (%) | 87,4 | 92,0 |  |  |
| <180 DDDs | N | 1361 | 102 | **0.62 [0.50;0.76]** | **<.0001** |
|  | Frequency (%) | 11,2 | 7,2 |  |  |
| ≥180 and <= 368 DDDs | N | 86 | 8 | NA | NA |
|  | Frequency (%) | 0,7 | 0,6 |  |  |
| > 368 DDDs | N | 88 | 3 | NA | NA |
|  | Frequency (%) | 0,7 | 0,2 |  |  |
| **NEFOPAM** | | | | | |
| 0 DDD | N | 11608 | 1329 | REF | REF |
|  | Frequency (%) | 95,3 | 93,9 |  |  |
| <180 DDDs | N | 564 | 87 | **1.35 [1.07;1.70]** | **.014** |
|  | Frequency (%) | 4,6 | 6,1 |  |  |
| ≥180 and <= 300 DDDs | N | 4 | 0 | NA | NA |
|  | Frequency (%) | 0,0 | 0,0 |  |  |
| > 300 DDDs | N | 4 | 0 | NA | NA |
|  | Frequency (%) | 0,0 | 0,0 |  |  |
| **NIFLUMIC ACID** | | | | | |
| 0 DDD | N | 11862 | 1386 | REF | REF |
|  | Frequency (%) | 97,4 | 97,9 |  |  |
| <180 DDDs | N | 315 | 30 | NA | NA |
|  | Frequency (%) | 2,6 | 2,1 |  |  |
| ≥180 and <= 390 DDDs | N | 2 | 0 | NA | NA |
|  | Frequency (%) | 0,0 | 0,0 |  |  |
| > 390 DDDs | N | 1 | 0 | NA | NA |
|  | Frequency (%) | 0,0 | 0,0 |  |  |
| **NIMESULIDE** | | | | | |
| 0 DDD | N | 11139 | 1305 | REF | REF |
|  | Frequency (%) | 91,5 | 92,2 |  |  |
| <180 DDDs | N | 985 | 107 | 0.93 [0.76;1.14] | .52 |
|  | Frequency (%) | 8,1 | 7,6 |  |  |
| ≥180 and <= 248 DDDs | N | 29 | 1 | NA | NA |
|  | Frequency (%) | 0,2 | 0,1 |  |  |
| > 248 DDDs | N | 27 | 3 | NA | NA |
|  | Frequency (%) | 0,2 | 0,2 |  |  |
| **OPIUM** | | | | | |
| 0 DDD | N | 11779 | 1375 | REF | REF |
|  | Frequency (%) | 96,7 | 97,1 |  |  |
| <180 DDDs | N | 363 | 37 | 0.87 [0.62;1.23] | .49 |
|  | Frequency (%) | 3,0 | 2,6 |  |  |
| ≥180 and <= 318 DDDs | N | 20 | 1 | NA | NA |
|  | Frequency (%) | 0,2 | 0,1 |  |  |
| > 318 DDDs | N | 18 | 3 | NA | NA |
|  | Frequency (%) | 0,1 | 0,2 |  |  |
| **OXETORONE** | | | | | |
| 0 DDD | N | 12125 | 1409 | REF | REF |
|  | Frequency (%) | 99,5 | 99,5 |  |  |
| <180 DDDs | N | 30 | 5 | NA | NA |
|  | Frequency (%) | 0,2 | 0,4 |  |  |
| ≥180 and <= 630 DDDs | N | 12 | 2 | NA | NA |
|  | Frequency (%) | 0,1 | 0,1 |  |  |
| > 630 DDDs | N | 13 | 0 | NA | NA |
|  | Frequency (%) | 0,1 | 0,0 |  |  |
| **OXYCODONE** | | | | | |
| 0 DDD | N | 11805 | 1368 | REF | REF |
|  | Frequency (%) | 96,9 | 96,6 |  |  |
| <180 DDDs | N | 234 | 34 | NA | NA |
|  | Frequency (%) | 1,9 | 2,4 |  |  |
| ≥180 and <= 728 DDDs | N | 71 | 7 | NA | NA |
|  | Frequency (%) | 0,6 | 0,5 |  |  |
| > 728 DDDs | N | 70 | 7 | NA | NA |
|  | Frequency (%) | 0,6 | 0,5 |  |  |
| **PARACETAMOL** | | | | | |
| 0 DDD | N | 3842 | 493 | REF | REF |
|  | Frequency (%) | 31,5 | 34,8 |  |  |
| <180 DDDs | N | 6064 | 720 | **1.75 [1.38-2.21]** | **<.0001** |
|  | Frequency (%) | 49,8 | 50,8 |  |  |
| ≥180 and <= 547 DDDs | N | 1123 | 97 | **2.51 [1.97-3.19]** | **<.0001** |
|  | Frequency (%) | 9,2 | 6,9 |  |  |
| > 547 DDDs | N | 1151 | 106 | **2.80 [2.20-3.55]** | **<.0001** |
|  | Frequency (%) | 9,4 | 7,5 |  |  |
| **PIROXICAM** | | | | | |
| 0 DDD | N | 10163 | 1185 | REF | REF |
|  | Frequency (%) | 83,4 | 83,7 |  |  |
| <180 DDDs | N | 1871 | 216 | 0.99 [0.85-1.15] | .95 |
|  | Frequency (%) | 15,4 | 15,3 |  |  |
| ≥180 and <= 318 DDDs | N | 71 | 10 | NA | NA |
|  | Frequency (%) | 0,6 | 0,7 |  |  |
| > 318 DDDs | N | 75 | 5 | NA | NA |
|  | Frequency (%) | 0,6 | 0,4 |  |  |
| **PREGABALINE** | | | | | |
| 0 DDD | N | 10609 | 1229 | REF | REF |
|  | Frequency (%) | 87,1 | 86,8 |  |  |
| <180 DDDs | N | 1141 | 133 | 1.00 [0.83-1.21] | 1.00 |
|  | Frequency (%) | 9,4 | 9,4 |  |  |
| ≥180 and <= 442 DDDs | N | 212 | 30 | NA | NA |
|  | Frequency (%) | 1,7 | 2,1 |  |  |
| > 442 DDDs | N | 218 | 24 | NA | NA |
|  | Frequency (%) | 1,8 | 1,7 |  |  |
| **RIZATRIPTAN** | | | | | |
| 0 DDD | N | 12162 | 1414 | REF | REF |
|  | Frequency (%) | 99,9 | 99,9 |  |  |
| <180 DDDs | N | 16 | 2 | NA | NA |
|  | Frequency (%) | 0,1 | 0,1 |  |  |
| ≥180 and <= 365 DDDs | N | 1 | 0 | NA | NA |
|  | Frequency (%) | 0,0 | 0,0 |  |  |
| > 365 DDDs | N | 1 | 0 | NA | NA |
|  | Frequency (%) | 0,0 | 0,0 |  |  |
| **SULINDAC** | | | | | |
| 0 DDD | N | 12143 | 1410 | REF | REF |
|  | Frequency (%) | 99,7 | 99,6 |  |  |
| <180 DDDs | N | 32 | 5 | NA | NA |
|  | Frequency (%) | 0,3 | 0,4 |  |  |
| ≥180 and <= 417 DDDs | N | 2 | 1 | NA | NA |
|  | Frequency (%) | 0,0 | 0,1 |  |  |
| > 417 DDDs | N | 3 | 0 | NA | NA |
|  | Frequency (%) | 0,0 | 0,0 |  |  |
| **SUMATRIPTAN** | | | | | |
| 0 DDD | N | 12153 | 1416 | REF | REF |
|  | Frequency (%) | 99,8 | 100,0 |  |  |
| <180 DDDs | N | 25 | 0 | NA | NA |
|  | Frequency (%) | 0,2 | 0,0 |  |  |
| ≥180 and <= 280 DDDs | N | 1 | 0 | NA | NA |
|  | Frequency (%) | 0,0 | 0,0 |  |  |
| > 280 DDDs | N | 1 | 0 | NA | NA |
|  | Frequency (%) | 0,0 | 0,0 |  |  |
| **TENOXICAM** | | | | | |
| 0 DDD | N | 11902 | 1387 | REF | REF |
|  | Frequency (%) | 97,7 | 98,0 |  |  |
| <180 DDDs | N | 263 | 24 | NA | NA |
|  | Frequency (%) | 2,2 | 1,7 |  |  |
| ≥180 and <= 360 DDDs | N | 8 | 2 | NA | NA |
|  | Frequency (%) | 0,1 | 0,1 |  |  |
| > 360 DDDs | N | 7 | 3 | NA | NA |
|  | Frequency (%) | 0,1 | 0,2 |  |  |
| **TIAPROFENIC ACID** | | | | | |
| 0 DDD | N | 11241 | 1341 | REF | REF |
|  | Frequency (%) | 92,3 | 94,7 |  |  |
| <180 DDDs | N | 929 | 74 | **0.67 [0.53-0.85]** | **0.001** |
|  | Frequency (%) | 7,6 | 5,2 |  |  |
| ≥180 and <= 428 DDDs | N | 5 | 1 | NA | NA |
|  | Frequency (%) | 0,0 | 0,1 |  |  |
| > 428 DDDs | N | 5 | 0 | NA | NA |
|  | Frequency (%) | 0,0 | 0,0 |  |  |
| **TRAMADOL** | | | | | |
| 0 DDD | N | 9116 | 1027 | REF | REF |
|  | Frequency (%) | 74,8 | 72,5 |  |  |
| <180 DDDs | N | 2517 | 320 | 1.12 [0.98-1.28] | 0.097 |
|  | Frequency (%) | 20,7 | 22,6 |  |  |
| ≥180 and <= 443 DDDs | N | 275 | 34 | NA | NA |
|  | Frequency (%) | 2,3 | 2,4 |  |  |
| > 443 DDDs | N | 272 | 35 | NA | NA |
|  | Frequency (%) | 2,2 | 2,5 |  |  |
| **VENLAFAXINE** | | | | | |
| 0 DDD | N | 11198 | 1335 | REF | REF |
|  | Frequency (%) | 91,9 | 94,3 |  |  |
| <180 DDDs | N | 656 | 50 | **0.64 [0.48-0.86]** | **0.0036** |
|  | Frequency (%) | 5,4 | 3,5 |  |  |
| ≥180 and <= 360 DDDs | N | 162 | 18 | NA | NA |
|  | Frequency (%) | 1,3 | 1,3 |  |  |
| > 360 DDDs | N | 164 | 13 | NA | NA |
|  | Frequency (%) | 1,3 | 0,9 |  |  |
| ** Median of the distribution of Defined Daily Doses (DDDs) in individuals with a DDDs ≥ 180 days*  *** NA; not applicable, minimum size not reached for odd ratio analysis* | | | | | |
